# Immune checkpoint receptor VISTA on immune cells is associated with expression of T-cell exhaustion marker TOX and worse prognosis in renal cell carcinoma with venous tumor thrombus

**DOI:** 10.1007/s00432-022-04329-y

**Published:** 2022-08-30

**Authors:** Łukasz Zapała, Michał Kunc, Sumit Sharma, Rafał Pęksa, Marta Popęda, Wojciech Biernat, Piotr Radziszewski

**Affiliations:** 1 Clinic of General, Oncological and Functional Urology, Medical University of Warsaw, Lindleya 4, 02-005 Warsaw, Poland; 2 Department of Pathomorphology, Medical University of Gdansk, 80-214 Gdańsk, Poland; 3 Laboratory of Translational Oncology, Intercollegiate Faculty of Biotechnology, Medical University of Gdańsk, 80-211 Gdańsk, Poland

**Keywords:** Renal cell carcinoma, Thrombus, PD-L1, VISTA, TOX, Tumor microenvironment

## Abstract

**Purpose:**

The study aimed to determine the expression of VISTA and TOX within venous tumor thrombus and primary clear cell renal cell carcinoma (ccRCC) and to assess their prognostic value.

**Methods:**

The study enrolled 82 patients with ccRCC and coexisting venous tumor thrombus treated radically from 2012 to 2019 in two tertiary centers. Tissue microarrays were prepared and stained with respective antibodies. The expression of markers was assessed separately on tumor cells (TCs) and/or tumor-associated immune cells (TAICs).

**Results:**

TOX expression was positively correlated with the percentage of VISTA-positive TAICs in venous thrombus (*p* = 0.011), but not in the primary tumor (*p* = 0.674). High TOX expression was associated with a higher percentage of PD-L1-positive TAICs in both compartments (*p* = 0.001, *p* = 0.011, respectively). Positive expression of VISTA on TAICs was associated with PD-L1 expression on TCs (*p* = 0.005) and TAICs (*p* = 0.004) in the primary tumor, and only with PD-L1 on TAICs in thrombus (*p* = 0.006). The presence of VISTA-positive TAICs in venous thrombus was significantly more common in females (*p* = 0.034), and positively correlated with metastases (*p* = 0.028), and tumor necrosis (*p* = 0.013). The cases with VISTA-positive TAICs in venous tumor thrombi had significantly shorter OS than VISTA-negative cases (*p* = 0.041).

**Conclusion:**

For the first time, we demonstrated the expression of VISTA- and TOX-positive TAICs in the venous tumor thrombus. We found the association between immune checkpoint receptors and *T* cell exhaustion markers in both tumor mass and venous thrombus. Finally, we demonstrated that abundance of VISTA-positive TAICs in venous tumor thrombus correlates with worse outcomes in ccRCC.

## Introduction

There is a growing interest in unveiling the fundamentals of the immune microenvironment of localized clear cell renal cell carcinoma (ccRCC), especially due to the need for the determination of novel biomarkers of possible prognostic properties (Ghatalia et al. 2019). Previous studies focused on tumor-associated immune cells (TAICs) and their role in cancer recurrence did not produce consistent results, e.g., Choueri et al. reported poorer prognosis in patients with increased CD8 + *T* cells (Choueiri et al. 2015), while Weiss et al. presented contradictory observations (Weiss et al. 2014). Thus, the prognostic value of tumor-infiltrating lymphocytes in ccRCC is yet to be established. Interestingly, in 10% of cases ccRCC produces venous tumor thrombus (Cao et al. 2019; Piotrowicz et al. 2015). Some research suggests cancer cells subclones in tumor thrombus are molecularly similar to the original RCC (Warsow et al. 2018). However, many authors pinpoint the discrepancies between the primary tumor and thrombus, including surrounding immune cells (Lopez et al. 2018; Zapala et al. 2022). Of note, venous thrombus has its unique microenvironment directly interacting with host peripheral blood (Cao et al. 2019). Hence, elucidating the molecular mechanisms of formation and propagation of tumor thrombus and the role of immune cells in these processes is of crucial importance (Niu et al. 2021).

Along with the introduction of novel immunotherapies, especially based on immune checkpoint inhibitors, the revolution in the treatment of advanced RCC is about to begin, setting new boundaries for adjuvant strategy (Heidegger et al. 2019). However, primary resistance and rare durable responses reflecting secondary resistance clearly explain the necessity for the establishment of prognostic and predictive factors related to the tumor immune microenvironment (Moreira et al. 2020). Heterogeneity is a major feature of the ccRCC microenvironment, while the special mechanisms of promotion and invasion are being engaged in tumor thrombus (Liss et al. 2019). Some subpopulations of TAICs may promote tumor growth and interfere with anti-tumor immune response (Mier 2019). One study reported that increased accumulation of programmed death-1 receptor (PD-1)-positive T cells was a determinant of worse survival (Senbabaoglu et al. 2016). Recently, we have found that the expression of programmed death-ligand 1 (PD-L1) expression on TAICs and/or tumor cells (TCs) in venous tumor thrombus was associated with significantly shorter overall survival (OS) (Zapala et al. 2022).

The next generation of immune checkpoints inhibitors emerges as a potential solution to overcome tumor resistance to anti-PD-L1/PD-1 pathway treatment. These include agents targeting the lymphocyte activation gene-3 (LAG-3), *T* cell immunoglobulin and mucin-domain containing-3 (TIM-3), and V-domain Ig suppressor of *T* cell activation (VISTA) (Qin et al. 2019; Franzin et al. 2020). Hong et al. reported that a poor response rate to PD-1 blockade may be attributed to the co-expression of other checkpoint receptors in the immunosuppressive tumor microenvironment, including VISTA (Hong et al. 2019). VISTA enforces inhibitory stimuli to the *T* cell population under physiological conditions, but an elevation of VISTA expression was found in prostate cancer patients treated with anti-CTLA-4 therapy, being a sign of growing resistance (Hong et al. 2022).

The other direction for future immunotherapy developments may be the studies on thymocyte selection-associated HMG BOX (TOX), that is *T* cell exhaustion marker (Kawashima et al. 2020). *T* cell exhaustion is a combination of loss of effector role (including production of cytokines) and an increase in inhibitory receptors (i.e., PD-1, TIM-3, CTLA-4 or LAG-3) under chronic antigen stimulation (Jiang et al. 2020). Thus, TOX may contribute to immune evasion in malignancy (Liang et al. 2021). Furthermore, TOX expression was inversely correlated with the efficacy of immunotherapy based on PD-1 blockade (Wang et al. 2019), so the combination of anti-TOX and immune checkpoint inhibitors may become a future therapeutic approach.

Hence, this study aimed to evaluate the expression of VISTA and TOX within venous tumor thrombus and primary ccRCC and to estimate their potency in predicting OS in this group of patients with RCC.

## Materials and methods

### Study group

The study group comprised 82 patients with clear cell RCC (ccRCC) and coexisting venous tumor thrombus treated with nephrectomy with/without cavotomy and thrombectomy in the years 2012–2019 in two tertiary referral urological centers. The standardized approach for kidney removal with/without cavotomy and thrombectomy was performed via lumbotomy or laparotomy. Using local databases we collected the following clinical data: age, sex, staging determined in CT or MRI scans of chest, abdomen, and pelvis according to the 2017 TNM classification system (Sobin et al. 2009), pathological assessment of the specimen including grading (according to WHO/ISUP), as well as the time of establishing diagnosis and death, and the last follow-up contact. Preoperative hematological data (number of neutrophils, platelets, lymphocytes, monocytes together with respective ratios: neutrophil to lymphocyte ratio, NLR; platelet to lymphocyte ratio, PLR; and lymphocyte to monocyte ratio, LMR) were retrieved from the local certified laboratories (FACS, Sysmex XM200, Sysmex Poland, Poland). No neoadjuvant treatment was carried out prior to surgery.

The study was performed under the local Ethics committee vote No. AKBE/72/2021 (Medical University of Warsaw). Informed consent was obtained from all the subjects involved in the study.

### Tissue microarrays

Tissue microarrays (TMAs) containing representative samples of the primary tumor mass and venous thrombus of ccRCC were prepared, as described previously (Zapala et al. 2022). Subsequently, the obtained sections were stained with anti-VISTA (clone D5L5T, 1:300 dilution, Cell Signaling) and anti-TOX (HPA018322, 1:200 dilution, Sigma Aldrich) antibodies. PD-L1 expression was evaluated, as reported previously (Zapala et al. 2022). Adequate positive controls (histologically normal tonsil and placenta) were incorporated in TMAs. The stainings were analyzed by two pathologists (MK and RP). The expression of the markers was assessed in TAICs (i.e., lymphocytes, macrophages, and granulocytes identified by morphology) and TCs separately in tumor mass and venous thrombus, and the percentage of positive cells in each compartment was estimated. The positive VISTA staining was defined as membranous and/or cytoplasmatic expression in at least 1% of cells, whereas TOX positivity was defined as nuclear staining in at least 10% of immune cells.

### Statistics

Statistical analyses were performed with Statistica 13 (RRID:SCR_014213, Tibco, Palo Alto, CA, USA) licensed to the Medical University of Gdańsk, and R Studio (Team RC 2013). The association between immunohistochemical markers and clinicopathological parameters was estimated with the chi-square test, Fisher’s exact test, and Wilcoxon test when applicable. Overall survival (OS) was defined as the time from diagnosis to the date of death due to any reason. “Survminer” package was employed to plot Kaplan–Meier curves and analyze the differences in survival between groups. Hazard ratios were calculated with Cox proportional hazard analysis. A *p*-value ≤ 0.05 was considered statistically significant.

## Results

### The baseline characteristics of the cohort

The baseline features of the study group were shown in (Table 1). Importantly, these were ccRCC cases with predominantly no sarcomatoid (92%) and/or rhabdoid (89%) features. In nearly 50% of specimens tumor necrosis was found. The majority of cases were staged as T3a (96,5%), while nodal involvement was present in 27% and distant metastases were observed in 28% of individuals.

**Table 1:** Basic characteristics of the study group

Feature	*N* (%)
Gender	
Male	45 (55)
Female	37 (45)
Age	Median: (IQR years) 66 (60–72)
Grade	
G2-G3	55 (67)
G4	27 (33)
Sarcomatoid features	
No	76 (93)
Yes	6 (7)
Rhabdoid features	
No	73 (89)
Yes	9 (11)
T	
3a	79 (96.5)
3b	1 (1.5)
3c	–
4	1 (1.5)
N	
0	67 (73)
1	15 (27)
M	
0	59 (72)
1	23 (28)
Tumor necrosis	
Present	38 (46)
Absent	44 (54)
Death during follow-up	
No	52 (63)
Yes	30 (37)

*IQR* interquartile range

### Expression of TOX and immune checkpoint receptors on TAICs

**Fig. 1:**
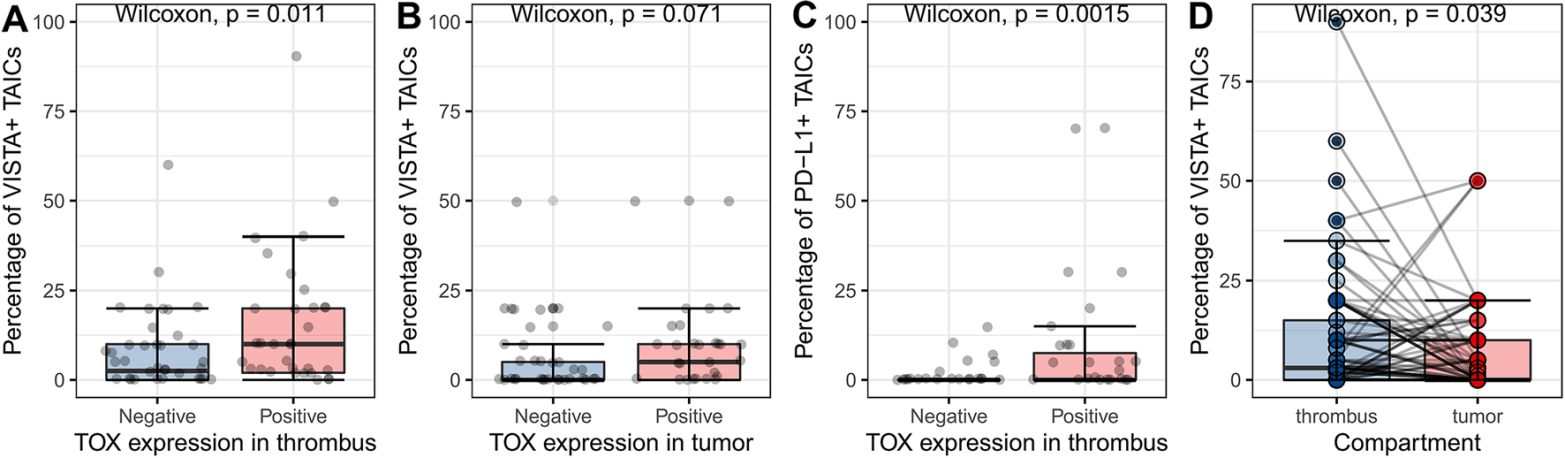
Expression of selected markers on tumor-associated immune cells in venous tumor thrombus and primary RCC. VISTA and TOX in tumor thrombus **A** and primary tumor **B**, PD-L1 and TOX in tumor thrombus **C**, comparison of VISTA expression between compartments **D** – lines connect corresponding cases. *PD-L1* programmed death-ligand 1, *TAICs* tumor-associated immune cells, *TOX* thymocyte selection-associated HMG BOX, *VISTA* V-domain Ig suppressor of *T* cell activation. The associations were estimated with the Wilcoxon test

The frequency of high nuclear TOX expression in both analyzed compartments was the same (*n* = 35, 48%), but many cases showed discrepant staining status in tumor mass and venous thrombus (*p* = 0.067, chi-square). TOX expression was positively correlated with the percentage of VISTA-positive immune cells in venous thrombus (*p* = 0.011, Wilcoxon test, Fig. 1A), but not in the primary tumor (*p* = 0.071, Wilcoxon test, Fig. 1B). Then, high TOX expression was associated with a higher percentage of PD-L1-positive immune cells in both compartments (*p* = 0.0015 in thrombus, *p* = 0.011 in primary tumor; Wilcoxon test, Fig. 1C). We did not detect the association between TOX and PD-L1 expression on tumor cells (*p* = 0.126 in thrombus, *p* = 0.227 in primary tumor; Wilcoxon test).

### Expression of VISTA and PDL-1 on TCs and TAICs

**Fig. 2:**
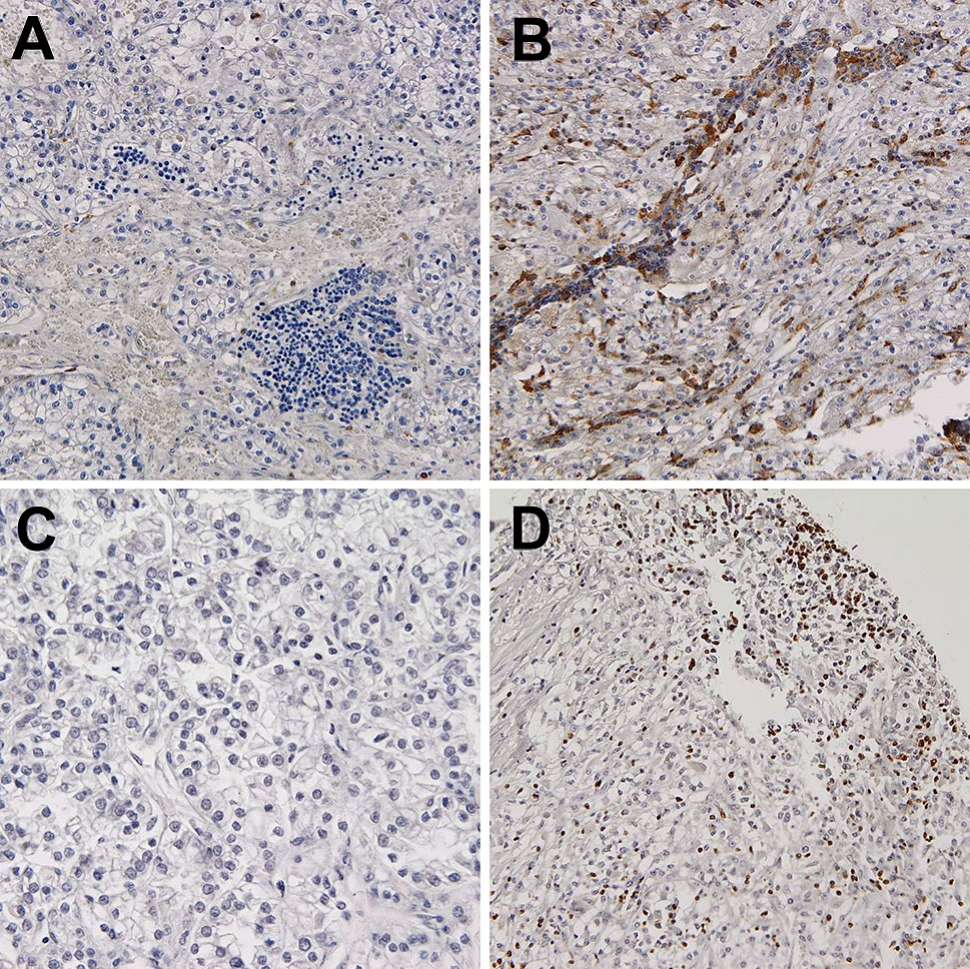
Representative examples of negative (**A**) and positive (**B**) VISTA staining in immune cells infiltrating ccRCC. Representative examples of negative (**C**) and positive nuclear TOX expression in > 10% of immune cells infiltrating ccRCC (**D**). *TOX* thymocyte selection-associated HMG BOX, *VISTA* V-domain Ig suppressor of *T* cell activation, *ccRCC* clear cell renal cell carcinoma

We did not observe VISTA expression on TCs in any case, whereas VISTA positivity in at least 1% of immune cells infiltrating venous tumor thrombus was detected in 48 out of 81 available cases (59%). The frequency of VISTA positivity was lower in primary tumors (*n* = 37, 46%) with borderline statistical significance (*p* = 0.099, chi-square). On the other hand, the percentage of VISTA-positive cells in venous tumor thrombi was significantly higher than in tumor masses (*p* = 0.039, Wilcoxon test, Fig. 1D). Positive expression of VISTA on TAICs was associated with PD-L1 expression on tumor cells (*p* = 0.005, chi-square) and immune cells (*p* = 0.004, chi-square) in the primary tumor, and only with PD-L1 on immune cells in thrombus (*p* = 0.006, chi-square). Representative examples of VISTA, PD-L1 and TOX staining in primary RCC and venous tumor thrombus were presented in (Fig. 2).

### Association between TOX and VISTA expression and clinicopathological variables

TOX expression was not significantly associated with any conventional clinicopathological variables (tumor size, nodal metastases, distant metastases, grade, and necrosis) (data not presented). On the contrary, the presence of VISTA-positive immune cells in venous thrombus was associated with gender (*p* = 0.034, chi-square), the presence of distant metastases (*p* = 0.028, chi-square), and tumor necrosis (*p* = 0.013, chi-square) (Table 2). In primary tumors, VISTA expression was associated with smaller tumor size (*p* = 0.028, chi-square).

**Table 2:** Association between VISTA expression in venous tumor thrombus/primary tumor and clinicopathological characteristics of RCC

Feature	VISTA in venous tumor thrombus			VISTA in primary tumor		
	Negative	Positive	*p*-value	Negative	Positive	*p*-value
Sex						
Female	10 (28)	26 (72)	**0.034**	20 (54)	17 (46)	0.959
Male	23 (51)	22 (49)		23 (53)	20 (47)	
Tumor size						
< 10 cm	21 (43)	28 (57)	0.631	21 (44)	27 (56)	**0.028**
> 10 cm	12 (37)	20 (63)		22 (69)	10 (31)	
Nodal metastases						
No	28 (42)	38 (58)	0.518	33 (51)	32 (49)	0.265
Yes	5 (33)	10 (67)		10 (67)	5 (33)	
Distant metastases						
No	28 (48)	30 (52)	**0.028**	33 (58)	24 (42)	0.241
Yes	5 (22)	18 (78)		13 (57)	10 (43)	
Grade						
2–3	14 (52)	13 (48)	0.150	15 (56)	12 (44)	0.817
4	19 (35)	35 (65)		28 (53)	25 (47)	
Tumor necrosis						
No	23 (53)	20 (47)	**0.013**	24 (57)	18 (43)	0.522
Yes	10 (26)	28 (74)		19 (50)	19 (50)	

*p*-values ≤ 0.05 were considered statistically significant (in bold)

*p*-values were calculated with the chi-square test

*VISTA* V-domain Ig suppressor of *T* cell activation. *P* values calculated with chi-square test

### Association between TOX and VISTA expression and markers of inflammatory response

**Fig. 3:**
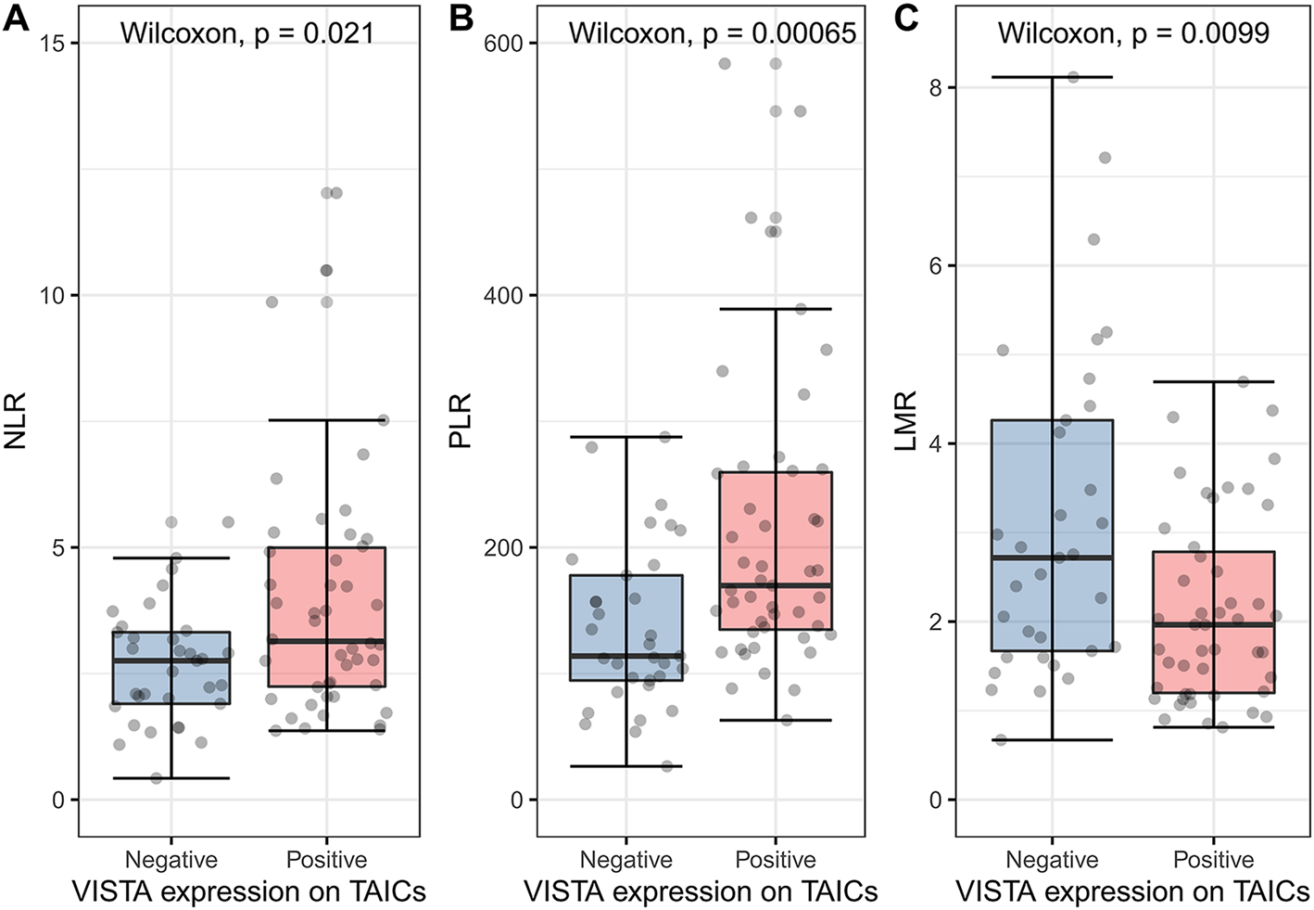
The association between VISTA expression on TAICs and systemic inflammatory markers: NLR (**A**), PLR (**B**), and LMR (**C**). *VISTA* V-domain Ig suppressor of *T* cell activation, *TAICs* tumor-associated immune cells, *NLR* neutrophil to lymphocyte ratio, *PLR* platelet to lymphocyte ratio,and *LMR* lymphocyte to monocyte ratio. The associations were estimated with the Wilcoxon test

To further investigate the relationship between local tumor and thrombus microenvironment and inflammatory response, we correlated TOX and VISTA expression on immune cells with the values of markers of systemic inflammation (Fig. 3). Higher NLR, PLR, and lower LMR corresponded with VISTA positivity in the tumor venous thrombus (*p* = 0.021, *p* < 0.001, and *p* = 0.01, respectively, Wilcoxon test), but not in the primary tumor (*p* = 0.345, *p* = 0.805, *p* = 0.798, respectively, Wilcoxon test). On the other hand, there was no difference in the levels of systemic inflammatory markers in TOX-positive and -negative cases in both compartments.

### Survival analysis

**Fig. 4:**
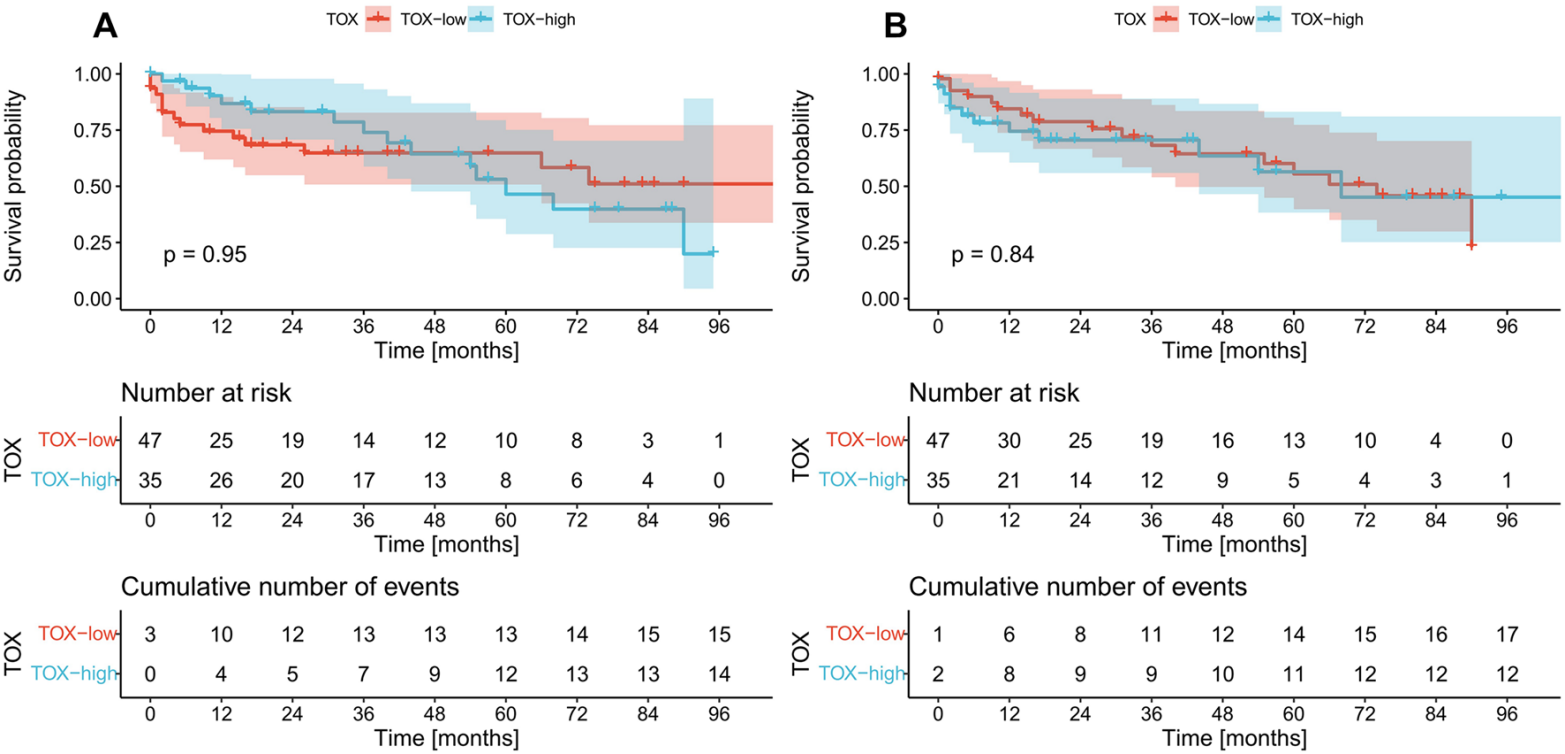
Kaplan–Meier curves for overall survival according to TOX expression on tumor-infiltrating immune cells in the venous tumor thrombus (**A**), and tumor mass (**B**). *p* values were calculated with the log-rank test. *Low* negative expression, *high* positive expression, *TOX* thymocyte selection-associated HMG BOX

**Fig. 5:**
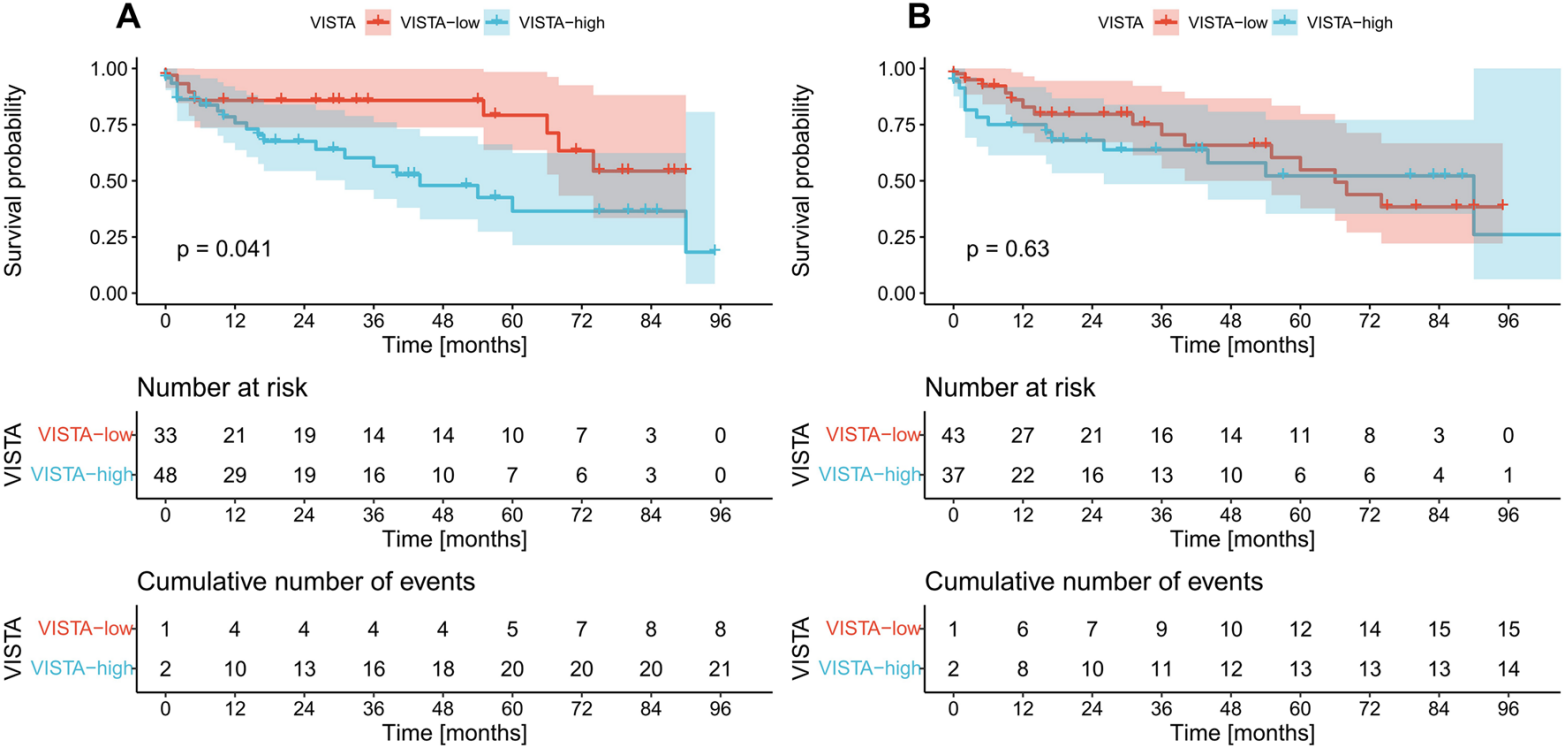
Kaplan–Meier curves for overall survival according to VISTA expression on tumor infiltrating immune cells in the venous tumor thrombus (**A**), and tumor mass (**B**). *p* values were calculated with the log-rank test. *Low* negative expression, *high* positive expression, *VISTA* V-domain Ig suppressor of *T* cell activation

In the next step, we assessed the prognostic significance of the analyzed markers. No differences in survival were identified between TOX positive and negative cases regardless of the studied compartment (Fig. 4A, B). Similarly, VISTA expression in primary tumors had no prognostic value (Fig. 5B). The cases with VISTA-positive immune cells in venous tumor thrombi had significantly shorter OS than VISTA-negative cases (*p* = 0.041, log-rank) with a hazard ratio of 2.3 (95% confidence interval 1.01–5.24, *p* = 0.046, univariate Cox regression) (Fig. 5A). However, this finding did not retain statistical significance in the multivariate analysis (data not presented).

## Discussion

In the current study, we demonstrated that VISTA receptor, located on TAICs, was associated with the expression of *T* cell exhaustion marker (TOX) in the venous tumor thrombus. Interestingly, VISTA-positive immune cells homing the thrombus, but not primary RCC, occurred to be the indicator of poor prognosis in terms of OS in the present cohort of patients.

Many authors emphasized the great impact of high lymphocyte infiltration within the center of the tumor on the anti-cancer response (Ghatalia et al. 2019; Moller et al. 2021; Gabrych et al. 2019). Here, we focused on the simultaneous expression of immune checkpoint receptors i.e. VISTA/PD-L1 and TOX on TAICs/TCs in different compartments. In RCC patients, VISTA is mainly found in CD45-positive cells of hematopoietic and myeloid origins in para- and tumorous tissues (Huang et al. 2020), and TOX is a marker of hematopoietic cells (Wang et al. 2019). In our cohort, no expression of VISTA on TCs in both primary tumor and venous tumor thrombus was noted. A recent paper by Hong et al. provided a unique analysis of VISTA expression and its function in the RCC microenvironment: even though ccRCC tumor cells expressed VISTA, a relatively low expression was observed (Hong et al. 2019). In their study, VISTA was found predominantly on intratumoral myeloid cells on contrary to PD-L1, which was expressed on CD45-negative cells.

It is hypothesized that the treatment with antibodies targeted to immune checkpoint inhibitors may reverse *T* cell exhaustion (Shin and Ribas 2015). In the murine RCC model (RENCA cells), VISTA expression correlated with poor CD8-positive *T* cell response, and the inhibition of VISTA restored the antitumoral activity (Hong et al. 2019). Liu et al. demonstrated in another murine model a distinct role of VISTA from the PD-1/PD-L1 signaling in the control of *T* cell activation (Liu et al. 2015). Furthermore, in the recent paper by Dornieden et al. on the profiling of lymphocytes homing peri- and tumorous tissues, it was reported that CD8-positive cells from RCC tumor tissue expressed simultaneously PD-1 and TOX (Dornieden et al. 2021). Another study demonstrated that inactivation of TOX may lead to a significant decrease in PD-1 or TIM-3, restoration of *T* cell function, and promotion of anticancer function of CD8-positive *T* cells (Liang et al. 2021). These findings correspond with the novel attitude towards the combination of systemic therapies based on immune checkpoint inhibitors and TOX-targeted therapies (Qin et al. 2019). In our study, there was a positive correlation between the presence of TOX and VISTA in TAICs, but only within the venous tumor thrombus, and VISTA-positive immune cells were found predominantly in this compartment. On the other hand, we found that positive TOX expression was present in PD-L1-positive immune cells homing both primary tumor and thrombus. Finally, positive VISTA expression on TAICs correlated with high PD-L1 expression on both TCs and TAICs in the renal tumor, but only with PD-L1 positive immune cells as for the thrombus. As a consequence, targeting a specific combination of inhibitory receptors may enable further personalization of systemic therapy. Recently, Chevrier et al. performed a study providing a deep insight into the profiling of immune cells infiltrates in ccRCC (Chevrier et al. 2017). Authors concluded that T CD8/PD-1-positive cells were characterized by simultaneous expression of inhibitory receptors. However, only a few PD-1-positive clusters were found to express TIM-3 or CTLA-4, making these molecules less promising candidates for future clinical trials in ccRCC (Chevrier et al. 2017). NCT04475523, NCT02812875, and NCT02671955 are ongoing VISTA-orientated clinical trials focused on the treatment of advanced solid tumors or lymphomas in adult patients (Hong et al. 2022).

Preliminary studies on VISTA in RCC seem to support the inhibitory properties of the receptor in the immune microenvironment of the tumor (Hong et al. 2019; Huang et al. 2020). Based on Tumor and Immune System Interaction Database, Huang et al. reported a positive correlation of VISTA and almost all critical immunomodulators (among them PD-1/PD-L1) in various cancers including RCC (Huang et al. 2020). However, as for the associations between VISTA and clinical features, the authors emphasized inconsistency in various tumors and did not confirm such links in the subanalyses of OS, tumor stages, or grading in RCC (Huang et al. 2020). However, in our microenvironment-oriented study of tumor thrombus, the expression of VISTA on TAICs was observed more frequently in female patients and correlated with distant metastases and tumor necrosis. Additionally, VISTA expression in primary tumors correlated with tumors < 10 cm in diameter.

Based on our previous paper on the application of blood-count-derived inflammatory biomarkers in venous tumor thrombus patients (Zapala et al. 2021), we aimed to the establishment of an association between these variables and the current receptors. Only VISTA expression in the venous tumor thrombus was associated with statistically significant changes in NLR, PLR and LMR values, while no correlations were found for TOX. Such an association was found in our previous studies on advanced germ cell tumors as for VISTA and PLR (Peksa et al. 2021). Some authors indicated that baseline blood count derivatives may be the prognostic tools in terms of the response of other highly immunogenic tumors, e.g., melanoma or non-small-cell lung carcinoma to nivolumab (Fujisawa et al. 2018; Bagley et al. 2017). Finally, increased NLR was found to be a statistically significant prognostic factor for poor OS in metastatic RCC patients on modern immunotherapy (Chen et al. 2021).

Taking into consideration the possible future application of TOX and VISTA in the prognostic models established for the RCC cases enrollment into clinical trials, one should consider their role in survival analyses. Here, we focused on the survival estimation in the subpopulation of RCC and venous tumor thrombus and found that the only prognostic value was ascribed to VISTA expression on immune cells within the thrombus compartment.

We are aware of a few limitations of our paper. To start with, it is a two-center retrospective study with the undeniable risk of bias. Using the well-recognized technique of TMAs, we did not evaluate properly the complete tumor sections. Finally, subprofiling of tumor-associated immune cells was not performed.

## Conclusions

For the first time, we demonstrated the expression of VISTA and TOX in the venous tumor thrombus. We found also the association between immune checkpoint receptors i.e., PD-L1 and VISTA and TOX, expressed mainly in TAICs, in the respective compartments. Finally, we confirmed additional characteristic features of tumor microenvironment of venous tumor thrombus.

## Data Availability

The data presented in this study are available on request from the corresponding authors.
